# Theoretical constraints on the precision and age range of rehydroxylation dating

**DOI:** 10.1098/rsos.140372

**Published:** 2015-04-01

**Authors:** V. J. Hare

**Affiliations:** Research Laboratory for Archaeology and the History of Art, University of Oxford, Dyson Perrins Building, South Parks Road, Oxford OX1 3QY, UK

**Keywords:** rehydroxylation, uncertainties, RHX dating, archaeological dating

## Abstract

Accurate and precise dating methods are of central importance to archaeology, palaeontology and earth science. This paper investigates the expected precision and age range of rehydroxylation dating, a recently proposed technique for fired clays. An expression for combined measurement uncertainty is presented, which takes into account all significant sources of experimental uncertainty. Numerical simulations are performed for comparison. Combined measurement uncertainties of approximately 5% with respect to the age of the ceramic should be possible given well-designed experiments. In this case, the most significant contribution to combined measurement uncertainty is from effective lifetime temperature. In addition, it is shown that precision should be acceptable for recently fired material (less than 1 year). Mismatch of balance resolution to sample mass results in large variation in combined relative uncertainties, which vary by four orders of magnitude (approx. 1–1160%) across recent experimental studies, rendering some recently reported dates meaningless. It is recommended that this ratio be less than 10^−6^ for a combined relative uncertainty of less than 1%. The age limits of the technique are set by the value of the rate constant and individual sample mineralogy. This theoretical framework should help future interlaboratory comparison as well as optimizing instrument design.

## 2. Context

There are several factors that determine the usefulness of an analytical dating method when applied to archaeological material. These may be the potential destruction of valuable material, cost or the length of time taken for analysis. However, of primary importance is the precision that the method is likely to afford, as well as the age range over which it is expected to be appropriate. Thus, in order to determine whether a new technique has any practical usefulness, the theoretical error limits need to be established [1].

Of course, the ultimate test of a dating technique is comparison with known-age materials, dated by either historical or stratigraphic considerations, or by an alternative method. However, in the absence of such comparisons, there will still be experimental and theoretical considerations which pose fundamental limitations. In radiocarbon dating, for example, the limits to measurement precision are predominantly determined by a combination of counting statistics and sample mass. In modern accelerator mass spectrometry, where ^14^C atoms are counted directly, this fundamental limit is ultimately set by the low abundance of radiocarbon (^14^C/^12^C in the range from 10^−12^ to 10^−15^). The number of radiocarbon atoms detected, *n*, follows a Poisson distribution, and measurement precision therefore goes as $1/\sqrt{n}$. The limit for a 12 ka old sample is typically approximately 25 a, corresponding to about 10^5^ atoms of radiocarbon, or a minimum mass of 10 μg C, not counting detector efficiency [2]. The older a sample is, the less ^14^C it contains, and consequently precise measurements become increasingly difficult at timescales more than 50 ka (roughly 8–9 half-lives).

Wilson *et al.* [3,4] proposed a new dating technique which has the potential to fill a long-standing gap in analytical methods of dating archaeological and historical fired clays. Rehydroxylation (RHX) dating developed from new insights [5] into the cause of long-term mass gain and expansion in bricks and tiles. A deeper understanding of these phenomena has only recently emerged (as reviewed in [6,7]). Both effects are a consequence of recombination of the ceramic matrix with atmospheric moisture (rehydroxylation). The proposal that long-term rehydroxylation is characterized by a (time)^1/4^ kinetic law [5,8] opened the possibility that the reaction might be used for archaeological dating.

The method has since been successfully applied to a small number of samples of archaeological pottery [4], but experimental difficulties have been reported by other authors [9–11] in applying the method to archaeological material. These may be related to the presence of organic interferents [4], and/or carbonates [12]. Consequently, one research priority is the development of appropriate chemical pre-treatment of archaeological material. Another important priority is determining the theoretical error limits of the technique, as well as an appropriate framework for combined measurement uncertainty. This would allow meaningful comparison between different studies. The aim of this paper is therefore to investigate the theoretical limits to rehydroxylation dating, as well as to quantify the combined measurement uncertainty associated with the technique.

## 3. Preliminaries: the dating equation

Rehydroxylation dating is based on accurate measurement of the hydroxyl content of fired clays, as well as accurate determination of the chemical kinetics of rehydroxylation. When aluminium layer silicates are fired, structural water (hydroxyl) is lost from the molecular lattice, along with several forms of more weakly bound water adsorbed on the pores and mineral interlayers. After firing, the collapsed layer silicates begin to recombine with atmospheric moisture. The hydration of the pore structures and interlayers is over within a couple of hours or days, but rehydroxylation is considerably slower, continuing over much longer timescales [8]. It is this reaction which is of relevance to archaeological dating. The power law model of Wilson *et al.* [5] describes the reaction kinetics:

3.1 $$y=\alpha (T)t^{1/4},$$

where *y* is the fractional hydroxyl mass gain owing to the rehydroxylation reaction, and *α*(*T*) is the rehydroxylation rate constant. The gravimetric method proposed by these authors [3,4] uses continuous mass measurements as a proxy for changes in hydroxyl content. In order to measure rehydroyxlation rate, a linear least-squares fit of the form *m*(*t*)=*m*_4_+*α*_m_*t*^1/4^ is performed on mass gain data once the gradient d*m*/d*t*^1/4^ has stabilized at constant temperature. Therefore, rehydroxylation is not measured directly, and as a consequence, other causes of changes in mass may contribute to the accuracy of the method. The factor of 1/4 in equation (3.1) is strongly suggestive of a process of anomalous diffusion [13,14], although the accurate kinetics of rehydroxylation in fired clays is an actively contested research area [11,15,16]. For the purposes of this study, we shall confine ourselves to the theoretical precision of the current model. Rearranging (3.1), the dating equation is given by

3.2 $$t_{a}={\left(\frac{y_{a}}{\alpha_{\mathrm{ELT}}}\right)}^{4},$$

where *t*_a_ is the age determination, *y*_a_ is the lifetime fractional OH^−^ mass gain owing to the RHX reaction, and *α*_ELT_ is the value of the RHX rate constant at the effective lifetime temperature. Measurements of *α* range between approximately 0.00018 and 0.00075 h^−1/4^ at common environmental temperatures [3,4]. Because the rate constant can only be measured at fixed temperatures in the laboratory, it has to be converted to that which the material would have experienced over its lifetime. The procedure, based on the Arrhenius relationship, is outlined in detail by Hall *et al.* [17]. In this method, *α*_ELT_ is calculated algorithmically as the fourth power mean over the entire lifetime temperature history of the ceramic, which is estimated from regional meteorological records.

In practice, equation (3.2) may also be formulated in terms of all measured quantities, which is necessary for determining a combined uncertainty in *t*_a_. For the purposes of this study, it will be convenient to write *α*_ELT_=(*α*_m_/*m*_4_)(*C*)^−1/4^, where

3.3 $$C=\exp\left[\left(\frac{E_{a}}{\text{R}}\right)\left(\frac{1}{T_{e}}-\frac{1}{T_{m}}\right)\right].$$

This converts the RHX constant *α*_m_, which is measured at laboratory temperature *T*_m_, to the RHX rate constant *α*_ELT_, at effective lifetime temperature *T*_e_. **R** is the universal gas constant, 8.314 J mol^−1^ K^−1^. *E*_a_ is the activation energy, and is obtained for each sample.

In addition, from the methodology of [4], we have that *y*_a_=(*m*_2_−*m*_4_)/*m*_4_. In essence, this states that the lifetime fractional OH^−^ mass gain is the measured difference between the as-received equilibrated mass, *m*_2_, and the dehydroxylated mass, *m*_4_, of the sample. Both measurements need to be conducted at precisely the same temperature and relative humidity in order for the contributions of weakly bound H_2_O to be negligible. It is important to note that this method of measuring lifetime OH^−^ will only be accurate if (i) dehydroxylation has proceeded to completion by reheating the sample to an appropriate temperature for sufficient time and (ii) RHX interferents such as calcite or organics are successfully removed from the sample [12,14]. RHX interferents may be any species which exhibit mass loss upon heating, causing inaccuracy in the measurement of *y*_a_, or those which are hygroscopic, causing inaccuracy in *α*_m_.

Under these two assumptions, the full calculation for RHX age is thus

3.4 $$t_{a}=\exp\left[\left(\frac{E_{a}}{\text{R}}\right)\left(\frac{1}{T_{e}}-\frac{1}{T_{m}}\right)\right]\times {\left(\frac{m_{2}-m_{4}}{\alpha_{m}}\right)}^{4}.$$

This contains five quantities (*m*_2_, *m*_4_, *α*_m_, *E*_a_, *T*_m_) which can be measured directly. It also contains one quantity (effective lifetime temperature, *T*_e_) which at present has to be estimated [17]. Alternatively, it might be possible to measure *T*_e_ independently using the same age sample method recently proposed by Moinester *et al.* [18]. Details of these methods are outside the scope of this paper: for our purposes, *T*_e_ is treated as known, with normally distributed errors.

Wilson *et al.* [4] assume that the primary component of uncertainty in RHX age is that of the measured RHX gradient, *α*_m_. In their study, they approach the problem by determining a relative uncertainty in the measured gradient, *u*(*α*_m_)/*α*_m_, by calculating

$$u(\alpha_{m})=\frac{s}{\sqrt{\left(n-2\right)}}\times \sqrt{\frac{n}{D}},$$

where *n* is the number of data points (*z*_*i*_) in a linear least-squares fit. *s* is the ℓ_2_ norm of the residuals:

$$s=\mathrm{sqrt}\left[\sum_{i=1}^{n}{\left(z_{i}-m_{4}-\alpha_{m}t_{i}^{1/4}\right)}^{2}\right],$$

and *D* is given as

$$D=n\sum_{i=1}^{n}z_{i}^{2}-{\left(\sum_{i=1}^{n}z_{i}\right)}^{2}.$$

Measurement uncertainty in RHX age (1*σ*) is taken as *u*(*α*_m_)/*α*_m_ multiplied by the RHX age (*t*_a_). This approach yielded uncertainties between 15 and 60 years for a range of dates, the earliest of which was 59 AD, and the most recent 1624 AD. A better approximation to measurement uncertainty would be larger. Indeed, although the most significant component of uncertainty in RHX ages is predicted to be that associated with the measured RHX gradient, Wilson *et al.* [4] point out that this approach does not represent a combined uncertainty, which should take into account measurement error in all quantities used to determine RHX age. This would include *m*_2_, *m*_4_, activation energy *E*_a_, as well as temperature terms. Moreover, it is also intuitive that this expression is inappropriate at recent timescales, when uncertainties in *m*_2_ and *m*_4_ must become significant. I shall explore two methods of better estimating combined uncertainty: a conventional Taylor-series propagation of error, as well as a Monte Carlo-type (MC) approach. Both are intended to be consistent with the Joint Committee for Guides in Metrology's *Guide to the expression of uncertainty in measurement* [19], as well as current terminology in the rehydroxylation dating literature. For the purposes of this study, I assume that the measurands are normally distributed, because the probability distributions (PDFs) of the underlying variables are not currently known. It should also be noted for future study that there is no *a priori* reason why these should be normally distributed, and that this case is most easily accommodated by MC approach.

## 4. Analytical expression of combined measurement uncertainty

Combined measurement uncertainty, *u*(*t*_a_), is conventionally approximated using a first order Taylor-series expansion about the sample variance of each measurand [19,20]. Applying this approach to equation (3.4) yields a partial expression for the square of the combined standard uncertainty, *u*^2^(*t*_a_), in RHX age *t*_a_ as

4.1 $$u^{2}(t_{a})=\sum_{i=1}^{6}{\left({\left.\frac{\partial t_{a}}{\partial x_{i}}\right|}_{{\bar{x}}_{1},\ldots ,{\bar{x}}_{6}}\right)}^{2}u^{2}(x_{i})+\cdots ,$$

where *x*_*i*_ are the six variables *m*_2_, *m*_4_, *α*_m_ *E*_a_, *T*_m_ and *T*_e_, each characterized by a mean value ${\bar{x}}_{i}$ and associated standard uncertainty *u*(*x*_*i*_). The square root of this expression represents a level of confidence in *t*_a_ of approximately 68% (1*σ*). An expanded uncertainty *U* may be calculated by multiplication with a coverage factor *k*:

4.2 $$U=ku(t_{a})$$

If *k*=2, *U* corresponds to a level of confidence of approximately 95%. This approach is only valid under the assumption that the variables are normally distributed, and input quantities are independent (i.e. covariance is negligible). In the case that input quantities are correlated, then the appropriate expression would be

4.3 $$u^{2}(t_{a})≃\sum_{i=1}^{6}{\left({\left.\frac{\partial t_{a}}{\partial x_{i}}\right|}_{{\bar{x}}_{1},\ldots ,{\bar{x}}_{6}}\right)}^{2}u^{2}(x_{i})+2\sum_{i=1}^{5}\sum_{j=1+1}^{6}\frac{\partial t_{a}}{\partial x_{i}}\frac{\partial t_{a}}{\partial x_{j}}u(x_{i},x_{j}),$$

where *u*(*x*_*i*_,*x*_*j*_) is the estimated covariance between the terms *x*_*i*_ and *x*_*j*_. Provisionally, it is assumed that covariance is negligible, and that all measurands are normally distributed. Thus, applying equation (4.1) to the full expression for RHX age (3.4) yields

4.4 $$\begin{array}{rl} u^{2}(t_{a}) & ≃{\left[\frac{4(C){\left(m_{2}-m_{4}\right)}^{3}}{\alpha_{m}^{4}}\right]}^{2}u^{2}(m_{2})+{\left[\frac{-4(C){\left(m_{2}-m_{4}\right)}^{3}}{\alpha_{m}^{4}}\right]}^{2}u^{2}(m_{4})\\ & \;+{\left[\frac{-4(C){\left(m_{2}-m_{4}\right)}^{4}}{\alpha_{m}^{5}}\right]}^{2}u^{2}(\alpha_{m})+{\left[\frac{(C){\left(m_{2}-m_{4}\right)}^{4}}{\text{R}\alpha_{m}^{4}}\left(\frac{1}{T_{e}}-\frac{1}{T_{m}}\right)\right]}^{2}u^{2}(E_{a})\\ & \;+{\left[\frac{-(C)E_{a}{\left(m_{2}-m_{4}\right)}^{4}}{\text{R}\alpha_{m}^{4}T_{e}^{2}}\right]}^{2}u^{2}(T_{e})+{\left[\frac{(C)E_{a}{\left(m_{2}-m_{4}\right)}^{4}}{\text{R}\alpha_{m}^{4}T_{m}^{2}}\right]}^{2}u^{2}(T_{m}). \end{array}$$

It is also possible to obtain a more concise expression for combined measurement uncertainty in RHX age as a function of the age itself. By equation (3.4), we have

4.5 $$t_{a}^{3/4}={\left(C\right)}^{3/4}\frac{{\left(m_{2}-m_{4}\right)}^{3}}{\alpha_{m}^{3}},$$

this can be substituted into equation (3.4) to yield

4.6 $$\begin{array}{rl} u^{2}(t_{a}) & ≃C^{1/2}{\left[\frac{4t_{a}^{3/4}}{\alpha_{m}}\right]}^{2}u^{2}(m_{2})+C^{1/2}{\left[\frac{-4t_{a}^{3/4}}{\alpha_{m}}\right]}^{2}u^{2}(m_{4})+{\left[\frac{-4t_{a}}{\alpha_{m}}\right]}^{2}u^{2}(\alpha_{m})\\ & \;+{\left[\frac{t_{a}}{\text{R}}\left(\frac{1}{T_{e}}-\frac{1}{T_{m}}\right)\right]}^{2}u^{2}(E_{a})+{\left[\frac{-E_{a}t_{a}}{\text{R}T_{e}^{2}}\right]}^{2}u^{2}(T_{e})+{\left[\frac{E_{a}t_{a}}{\text{R}T_{m}^{2}}\right]}^{2}u^{2}(T_{m}). \end{array}$$

## 5. Limits to the precision of the technique

### 5.1 Considering uncertainties in *m*_2_, *m*_4_ and *α*_m_

Equation (4.6) may also be rearranged to yield an expression for relative uncertainty in the RHX age determination. First, we consider the effects of uncertainty in *m*_2_, *m*_4_ and *α*_m_, in the absence of temperature terms. If it is assumed that the RHX rate constant *α*_m_ is measured at the effective lifetime temperature, then *T*_m_=*T*_e_, and *C* (equation (3.3)) is unity. In this case, we have

5.1 $$u(t_{a})\geq \left(\frac{4}{\alpha_{m}}\right)\times \sqrt{\left(t_{a}^{3/2})(u^{2}(m_{2})+u^{2}(m_{4}))+t_{a}^{2}u^{2}(\alpha_{m}\right)}.$$

Because $t_{a}^{3/2}=t_{a}^{2}t_{a}^{-1/2}$, equation (5.1) may be manipulated to yield the relative uncertainty in RHX age:

5.2 $$\frac{u(t_{a})}{|t_{a}|}\geq \left(\frac{4}{\alpha_{m}}\right)\times \sqrt{\left(u^{2}(m_{2})+u^{2}(m_{4}))t_{a}^{-1/2}+u^{2}(\alpha_{m}\right)},\;t_{a}\neq 0.$$

These expressions are of some importance, because they predict the range over which the RHX method should be useful. Equation (5.1) predicts that when *t*_a_ is small the first term under the square root in the above expression will dominate, and consequently the relative error in RHX dates scales as 1/*t*^1/4^_a_. Here, the transition point is defined by $t_{\mathrm{trans}}≃([u^{2}(m_{2})+u^{2}(m_{4})]/u{\left(\alpha_{m}^{2})\right)}^{2}$. Conversely, at *t*_a_≫*t*_trans_, the first term under the square root will be negligible, and therefore the relative uncertainty will be approximately constant with time:

5.3 $$\frac{u(t_{a})}{|t_{a}|}\approx \frac{4u(\alpha_{m})}{\alpha_{m}}.$$

It may be noted that the above expression is similar to that used by Wilson *et al*. [4], with the exception of multiplication by a factor of 4. For practical purposes of dating, the linear increase in uncertainty at large times is very slow, so that for typical values of *α*_m_ reported by these authors we can expect a minimum relative uncertainty of approximately 0.5%, which translates to an increase of 10 years in uncertainty every 2000 years or so. However, by (5.2), the combined relative uncertainty of very *recent* material should increase sharply according to the power law (for *t*_a_≪*t*_trans_). If C is unity, *α*_ELT_=*α*_m_/*m*_4_, and so an approximation to the relative percentage uncertainty at large *t*_a_ may also be written:

5.4 $$\frac{u(t_{a})}{|t_{a}|}\times 100\approx \left(\frac{400}{m_{4}}\right)\frac{u(\alpha_{m})}{\alpha_{\mathrm{ELT}}}.$$

Although it must be stressed that this equation is an approximation, it is nevertheless a useful expression to compare baseline uncertainty in RHX age determination, as well as to optimize the design of future experiments. Because the above expression neatly combines several concepts in rehydroxylation dating, it will be convenient to assign to it a symbol, ‘*ϕ*’:

5.5 $$\phi \equiv \left(\frac{400}{m_{4}}\right)\frac{u(\alpha_{m})}{\alpha_{\mathrm{ELT}}}.$$

In practical terms, this means that combined relative uncertainty is decreased by:

— increasing the resolution of the balance/microbalance, which decreases *u*(*α*_m_),
— samples with higher *α*_ELT_, which may be those originally fired at lower temperatures, and/or those which experienced higher effective lifetime temperatures,
— increasing the total sample mass selected for the dating procedure, thereby increasing *m*_4_, and
— increasing either the number of points of the microbalance data collection and/or the time interval over which data are acquired, which decreases *u*(*α*_m_).

Table 1 compares the theoretical minimum relative uncertainty in RHX ages (‘*ϕ*’) across a range of recent experimental publications. By using a nominal *α*_ELT_ of 0.00023 h^−1/4^ (in effect the same ‘virtual’ material used across different investigations, with identical lifetime temperature histories), it is possible to compare the minimum theoretical precision afforded by each experimental system. Where data for *m*_4_ and *u*(*α*_m_) are not directly reported, we make two reasonable assumptions: (i) that *m*_4_ is approximately equal to the total mass of the sample used in the experiment, and (ii) that *u*(*α*_m_) is essentially determined by the resolution of the balance/microbalance. The first assumption is motivated by the fact that structural OH^−^ can only take up a stoichiometric maximum of about 13.95% [24] of the total sample mass (in the case of kaolinite, for example). The second assumption is made for the comparison of *ϕ* to be consistent between studies. *u*(*α*_m_) is taken as one order of magnitude greater than the balance resolution in each study, although Moinester *et al*. [18] calculate that magnitudes of *u*(*α*_m_) approach the quoted balance resolution if repeated measurements are performed using a microbalance of 0.1 μg resolution over a 16 h period. In effect, the values in table 1 are therefore conservative. Note that decreasing *u*(*α*_m_) by one order of magnitude will decrease the estimates of *ϕ*, but uniformly across studies. It will be seen that there is a considerable range in relative uncertainty, from 1160% to less than 1%, which is primarily the result of a mismatch between balance resolution and sample mass. We suggest that this effect ultimately sets a fundamental limit to the precision achievable in the dating technique. Consequently, it is recommended that the resolution:mass ratio should be approximately 10^−6^ or less for a minimum combined relative uncertainty of 1%.

**Table 1.** Order of magnitude estimates of minimum relative uncertainty in RHX age, *ϕ*, between recent experimental studies of rehydroxylation in archaeological ceramics. Values of *u*(*α*_m_) are conservative estimates, and are one order of magnitude greater than the quoted balance/microbalance resolution. Note that *ϕ* is a minimum estimate of *u*(*t*_a_) in the special case that *T*_e_=*T*_m_, and *u*(*T*_e_) and *u*(*T*_m_) are both negligible.

	*α*_ELT_ (h^−1/4^)	*u*(*α*_m_) (g h^−1/4^)	*m*_4_ (g)	*ϕ*%
Barrett [21]	0.00023	0.0001	40	4.4
Bowen *et al.*[15]	0.00023	0.001	32	54.3
Burakov *et al.*[10]	0.00023	0.01	8.5	1160
Drelich *et al.*[22]	0.00023	0.00001	1.5	11.6
Hare [23]	0.00023	0.001	60	29.0
Le Goff *et al.*[9]	0.00023	0.00001	3	5.8
Wilson *et al.*[4]	0.00023	0.000001	2	0.9

### 5.2 Considering uncertainties in temperature

Equation (4.6) may also be used to derive expressions for the contributions of temperature terms to the combined relative uncertainty in the case that *t*_a_≠0 and *T*_e_≠*T*_m_:

5.6 $$\begin{array}{rl} \frac{u(t_{a})}{|t_{a}|} & \geq \frac{E_{a}u(T_{e})}{\left(\text{R}T_{e}^{2}\right)}, \end{array}$$

5.7 $$\begin{array}{rl} \frac{u(t_{a})}{|t_{a}|} & \geq \frac{E_{a}u(T_{m})}{\left(\text{R}T_{m}^{2}\right)} \end{array}$$

5.8 $$\begin{array}{rl} \;\text{and}\;\frac{u(t_{a})}{|t_{a}|} & \geq \frac{u(E_{a})}{\text{R}}\left|\frac{1}{T_{e}}-\frac{1}{T_{m}}\right|. \end{array}$$

**Figure 1.**
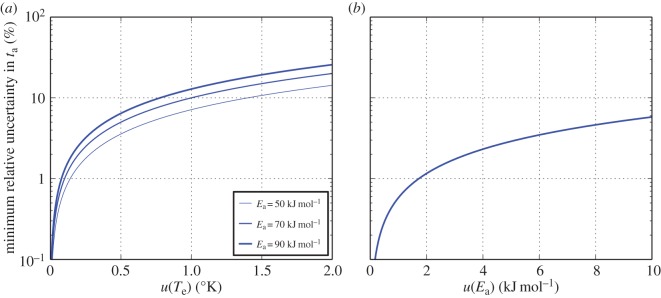
Minimum uncertainty in RHX age *t*_a_ owing to uncertainties in (*a*) effective lifetime temperature (equation (5.6)) and (*b*) activation energy (equation (5.8)). Three different activation energies are shown in (*a*), within a range of those commonly reported for the RHX reaction [3,4].

The right-hand term in (5.6) is the minimum relative uncertainty in RHX age due to uncertainty in lifetime temperature history, which, reassuringly, is the same form as the error term presented in [17]. If it is assumed that the uncertainty in effective lifetime temperature must be at least around 0.1°C, then the contribution of this term to the relative uncertainty in *t*_a_ is at minimum 0.55%, given a likely effective lifetime temperature of around 13°C and an activation energy *E*_a_ for the rehydroxylation reaction of around 70 kJ mol^−1^. This translates to about half a year in 100 years, 5.5 years in 1000 years, etc. (effectively 1*σ*). This is the same order of magnitude as contributions of uncertainties in *m*_2_, *m*_4_ and *α*_m_, in the case that the resolution:mass ratio is less than 10^−6^. The magnitude of this effect is also in agreement with simulations recently performed by Moinester *et al.* (see table I in [18]). The other two expressions (5.7) and (5.8) relate to *u*(*T*_m_) and *u*(*E*_a_), which are less significant contributions to combined relative uncertainty. This effect is evident in figure 1, which shows the minimum contributions of uncertainty in effective lifetime temperature (figure 1*a*) and uncertainty in activation energy (figure 1*b*). Uncertainty in effective lifetime temperature is unknown in the sense that it depends on the thermal history of the ceramic object, and is largely out of the control of the experimentalist. It is also likely to be much greater than 0.1°C. On the other hand, equations (5.7) and (5.8) suggest that the contributions of both *u*(*T*_m_) and *u*(*E*_a_) may be reduced by changes to experimental design: either greater thermal stability in an RHX experiment, or setting the measurement temperature as closely as possible to the effective lifetime temperature (*T*_m_≈*T*_e_).

### 5.3 Comparison with a simple Monte Carlo approach

**Figure 2.**
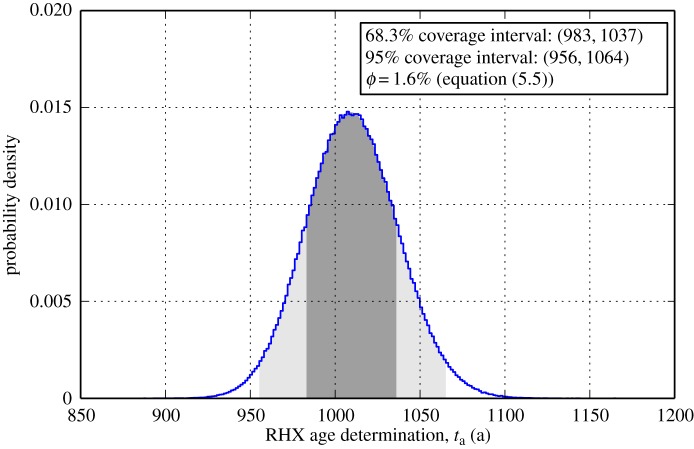
Probability density plot for a simulated RHX age determination (*N*=10^6^ histories) with parameters $m_{4}∼N(1.412509,1\times 10^{-5})\;g$, $m_{2}∼N(1.426100,1\times 10^{-5})\;g$, $\alpha_{m}∼N(0.0002562,1\times 10^{-6})\;g\;h^{-1/4}$, $E_{a}∼N(70,2)\;\mathrm{kJ}\;{\mathrm{mol}}^{-1}$, $T_{m}∼N{\left(287.0,0.1\right)}^{\circ }K$, $T_{e}∼N{\left(286.0,0.18\right)}^{\circ }K$. Dark grey and light grey regions represent coverage intervals at 68.3% and 95% probability levels, respectively. The RHX age determination is the age of the ceramic since original firing, in years.

The first-order Taylor-series approach has been presented because it provides useful analytical expressions for the minimum contributions of component uncertainties as well as optimization of experimental design. However, there are undoubtedly more powerful methods which are better suited to the task of estimating combined uncertainty. One such approach is to use an MC method (see [25] for an introduction). The rationale is to assume underlying PDFs for each of the continuous random variables *m*_2_, *m*_4_, *α*_m_, *E*_a_, *T*_e_ and *T*_m_. We seek the PDF of *t*_a_, which by MC method is approximated by $\bar{t_{a}}=(1/N)\sum_{i=1}^{N}t_{a}({\text{x}}_{\text{i}})$, where *N* is the number of samples (histories) of **x**_**i**_ drawn from the joint PDF. In the simplest case, this PDF can be approximated by assuming that the measurands are described by independent normal distributions of known mean and variance, i.e.

$${\text{x}}_{\text{i}}∼N(\mu_{i},\sigma_{i}),$$

and sampling several hundred thousand times over the RHX age equation (3.4). As in the previous section, we have assumed normal distributions and negligible covariance. A theoretical rehydroxylation age PDF is shown in figure 2. The parameters *μ*_*i*_ are the same orders of magnitude as those in Wilson *et al.* [4], as are *σ*_*i*_ for *E*_a_ and *T*_m_. However, the parameters *σ*_*i*_ for *m*_2_ and *m*_4_ are two orders of magnitude greater than those which might be obtained using a microbalance of 0.1 μg resolution. In part, this is because other factors might contribute to these uncertainties in *m*_2_ and *m*_4_. These include (i) issues of mass loss due to sample transfer between the measurements of *m*_2_ and *m*_4_, as well as (ii) issues of mass loss due to interferents. In addition, *σ*_*i*_ for *α*_m_ is taken as one order of magnitude higher than the microbalance resolution. Therefore, *σ*_*i*_ are conservative estimates for these measurands. Because uncertainties for *T*_e_ are not reported in Wilson *et al.* [4], I follow Moinester *et al.* [18] by taking *σ*_*i*_ for *T*_e_ as 0.18°C. The MC estimate of rehydroxylation age is $\bar{t_{a}}=1010\pm 27\;a$, which is in good agreement with the analytical expression from the first-order Taylor series (*u*(*t*_a_), equation (4.4)). Combined relative uncertainty in this RHX age determination by the MC method is therefore 2.7% (68.3% probability level). The minimum relative uncertainty associated with non-temperature terms (*ϕ*) is approximately 1.6%.

**Figure 3.**
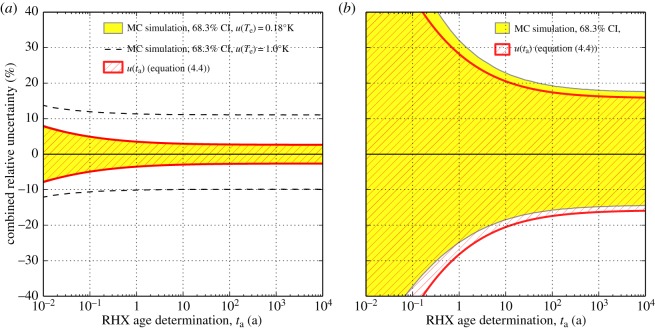
Comparison of uncertainties estimated by a Monte Carlo method (yellow regions, representing a coverage interval at 68.3% confidence) with the first-order Taylor series approach (equation (4.4)) for a theoretical sample with constant parameters $E_{a}∼N(70,2)\;\mathrm{kJ}\;{\mathrm{mol}}^{-1}$, $T_{m}∼N{\left(287.0,0.1\right)}^{\circ }K$. (*a*) Represents a scenario with parameters identical to figure 2 and based on sample S1 in Wilson *et al.* [4], with $m_{4}∼N(1.412509,1\times 10^{-5})\;g$, $\alpha_{m}∼N(0.0002562,1\times 10^{-6})\;g\;h^{-1/4}$ and $T_{e}∼N{\left(286.0,0.18\right)}^{\circ }K$. For comparison, dashed black lines indicate uncertainty in effective lifetime temperature of 1°K. (*b*) Shows a scenario where uncertainties in *α*_m_, *m*_4_ and *m*_2_ are all increased by one order of magnitude relative to their level in (*a*).

If repeated simulations are performed sequentially for increasing *m*_2_ (which increases *t*_a_ by increasing the amount of OH^−^ recombined with the matrix), then combined uncertainty may be plotted as a function of RHX age. In figure 3, these are plotted as the percentage uncertainty (negative and positive) of the RHX age against a range of possible ages. The MC propagated uncertainty, which represents a coverage interval at 68.3% probability, is compared with the Taylor-series approach (red lines) for a hypothetical ceramic. The two methods are generally in good agreement. Figure 3*a* has the same parameters as the simulation in figure 2, which are based on sample S1 in Wilson *et al.* [4]. An uncertainty of 0.18°K in effective lifetime temperature might be unrealistic, particularly in the case of archaeological pottery. The reasons include that

— the frequency of use of pottery for cooking is largely unknown,
— it may be difficult to estimate burial rates,
— regional meteorological records will not correspond directly to the temperature of the ceramic, and specific heat capacities may need to be accounted for, and
— other uncertainties may enter the numerical method of determining *α*_ELT_ and *T*_e_, which we have so far not considered.

For comparison, we include a simulation with uncertainty in effective lifetime temperature of 1°K in figure 3*a* (dashed black lines) which shows that combined relative uncertainties increase to approximately 10%. Both simulations therefore show that it should be possible to determine the age of recently fired clay (under 1 year old, for instance) with acceptable combined relative uncertainty. While of limited archaeological significance, this may be of importance as a test of the accuracy of the technique. A clay might be fired in the laboratory, kept under constant controlled temperature (hence *T*_e_ could be determined with high accuracy), and an RHX age determination of acceptable precision obtained within a few months of the original firing. In contrast, radiocarbon or luminescence methods do not offer the possibility of such precision over very recent timescales. However, such a test would present considerable practical challenges if the current gravimentric method is to be used. It would require precise measurement of the equilibrated mass *m*_2_ over and above the underlying mass gain due to rehydroxylation, the latter process also being measurable in the first few weeks of firing. It should be added that the stabilization of *m*_2_ is a very important condition, and one which has proved problematic in our own laboratory [23] as well as elsewhere [9,11].

Figure 3*b* shows the effect of increasing uncertainty in *α*_m_ by one order of magnitude from 10^−6^ to 10^−5^ g h^−1/4^, and uncertainties in *m*_2_ and m_4_ from 10^−5^ to 10^−4^ g. The effect can clearly be seen as a broadening of the coverage interval over all RHX age determinations, which are non-symmetric in the case of the MC method. Such large combined uncertainties might render an RHX age determination meaningless. Relative uncertainty in the masses *m*_2_ and *m*_4_ should be less than 10^−5^ for a combined uncertainty in *t*_a_ of less than 10%. Such large uncertainties in *m*_2_ and *m*_4_ may be caused by inappropriate choice of sample mass and/or balance resolution, or necessitated by the presence of RHX interferents.

## 6. The age limits of the technique

An expression for the age limits of the technique is now suggested. I start with the assumption that there are a finite number of sites at which OH^−^ may recombine with the ceramic matrix. It follows that there must be a point at which the rehydroxylation reaction stops, i.e. all available OH^−^ sites are filled, and *y*_a_=*y*_max_. Beyond this point, an RHX age determination must be regarded as a minimum age estimate. Assuming that *t*^1/4^ kinetics are obeyed for the entire lifetime of the fired clay, then equation (3.2) suggests

6.1 $$t_{\max}={\left(\frac{y_{\max}}{\alpha_{\mathrm{ELT}}}\right)}^{4},$$

where *y*_max_ is given by (*m*_max_−*m*_4_)/*m*_4_. For pure kaolinite, the dehydroxylation reaction is

$$2{\mathrm{Al}}_{2}{\mathrm{Si}}_{2}O_{5}{\left(\mathrm{OH}\right)}_{4}\to 2{\mathrm{Al}}_{2}{\mathrm{Si}}_{2}O_{7}+4H_{2}O.$$

**Figure 4.**
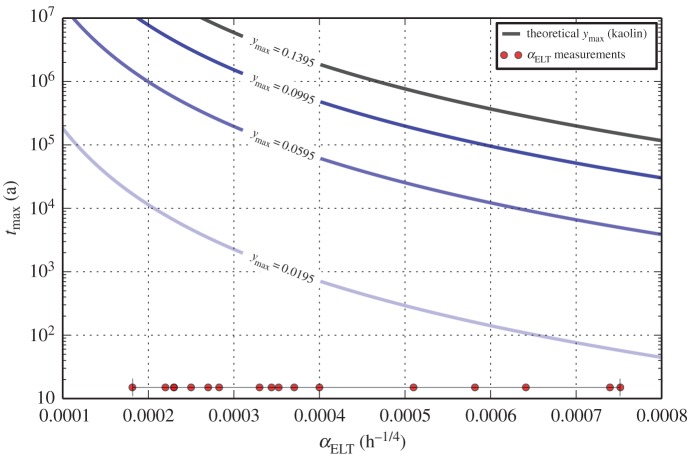
The age limits of rehydroxylation dating. Dark blue curves are simulations of constant *y*_max_ plotted for increasing *α*_ELT_, and light-blue-shaded areas indicate 68.3% coverage intervals estimated by MC method (parameters same as those in figure 2). Lowering *T*_e_ shifts the curves upwards, but only slightly. The black curve represents the maximum age limits for pure kaolinite (assuming the dehydroxylation reaction is fully reversible). For archaeological samples, *y*_max_ is likely to be lower. The range of measurements of *α*_ELT_ [3,4,23] is shown along the *x*-axis.

If dehydroxylation is fully reversible, then one might expect by stoichiometry that OH^−^ will form a maximum of 13.95% [24] of the final rehydroxylated matrix, and consequently *y*_max_=0.1395. However, archaeological clays are complex mixtures of different clay minerals, and are rarely pure kaolinite. The value of *y*_max_ is likely to be lower, and unique to each archaeological ceramic. The degree of lattice substitution and intercalation, particularly if dioctahedral 2 : 1 layer clay minerals are present, should lower *y*_max_. Additionally, dehydroxylation will not be fully reversible in highly fired ceramics. These should have lower *y*_max_ owing to the formation of high-temperature phases (spinel/mullite in the case of kaolinite). Lower *y*_max_ will reduce the range over which the dating technique would be useful, because there are less sites at which OH^−^ may recombine with the matrix, and the rehydroxylation reaction will be over sooner. On the other hand, lower *α*_ELT_ (either from low *T*_e_ and/or high original firing temperatures) should slow the reaction, increasing the age range. The two effects are shown in figure 4, where isolines of *y*_max_ are plotted against the known range of *α*_ELT_. The range of measured values of *α*_ELT_ [3,4] is also shown. For pure kaolinite, fired at relatively low temperatures, dehydroxylation might be fully reversible, and *t*_max_ should be greater than 100 000 years over the entire range of *α*_ELT_. This is clearly far beyond the range of ceramic technology. However, for a sample with very low *y*_max_ (in the range of 2% OH^−^ by mass), *t*_max_ is considerably less, and lies between 10 000 and 1000 years for *α*_ELT_ between 0.0002 and 0.0004 h^−1/4^. In practice, *y*_max_ is difficult to measure. However, it may be possible to speed the RHX reaction to completion by steam autoclave experiments (see fig. 3*b* in [26]). *y*_max_ could then be determined by differential thermal analysis. However, this may involve mechanisms that differ from rehydroxylation under standard conditions. As a complementary approach, it should be possible to measure *t*_max_ for each sample directly by extension of the current method for determination of activation energy [4,17]. It is noted that Hall *et al.* [17] identify the conventional solid-state rate constant *k*(*T*) [27] as (*α*(*T*)/*y*_max_)^4^. Because *k*(*T*) is also described by Arrhenius' equation *k*(*T*)=Aexp[−*E*_a_/(**R***T*)], it is identified that

6.2 $$t_{\max}=A^{-1}\exp\left[\frac{E_{a}}{\left(\text{R}T_{\text{e}}\right)}\right].$$

*A* is the pre-exponential (frequency) factor in the Arrhenius equation, which along with *E*_a_ is measured experimentally by determining the *y*-intercept and gradient of a plot of ln⁡(k) versus 1/*T* for several different temperatures. Note that for a first order reaction, *A* has dimensions of [time^−1^], as expected. Therefore, *t*_max_ may be determined if *T*_e_ and *A* (or *y*_max_) are known. We briefly note that this expression determines the ‘lifetime’ of fired clays, and should be of some importance to studies of the long-term stability of structural ceramics. Additionally, it could provide a useful test of the assumption that the reaction is first order, or that *t*^1/4^ kinetics are obeyed throughout the lifetime of the ceramic.

## 7. Conclusion

Two methods were presented for determining combined uncertainty in rehydroxylation dating, which reveal fundamental limits to the technique. Minimum uncertainties vary across four orders of magnitude (approx. 1–1160%) in recent experimental studies of rehydroxylation in archaeological ceramics, which may partly explain the failure of certain studies to achieve meaningful results. The ratio of balance resolution to sample mass emerges as a significant effect. It is recommended that this ratio be less than 10^−6^ to achieve a minimum level of uncertainty comparable to that in Wilson *et al.* [4]. In addition, propagation of uncertainty by MC method shows that it should be possible to obtain an RHX age determination on very recent material (less than 1 year) with acceptable precision (less than 10%). A new expression for the age limit of the technique is presented which depends on both *y*_max_, which is likely to be set by clay mineralogy, and *α*_ELT_, which is set by a combination of original firing temperature, mineralogy and *T*_e_. In most cases, the technique should provide acceptable precision for ceramics of any age. The exceptions would be very highly fired clay, such as porcelain, where either *α*_ELT_ or *y*_max_ is very low. In the absence of other information, we have assumed all measurands are normally distributed, with negligible covariance. This is unlikely to be the case, and more refinements are probably necessary. It should be noted that we have so far not discussed other important systematic effects on measurement error. In particular, the effects of RHX interferents need to be investigated, and the development of appropriate pre-treatments should be a research priority if the method is to be both robust and accurate. Recent work [28] on the removal of organic carbon has shown promise. The ultimate test of a dating technique will be comparison with alternative dating methods. Many more studies are needed in both regards.

## Data accessibility

MATLAB (R2014a) code used for simulations of combined uncertainties may be found in the Dryad Digital Repository (doi:10.5061/dryad.q4t65), along with supporting data used in figure 4.
